# Biodiversity loss and turnover in alternative states in the Mediterranean Sea: a case study on meiofauna

**DOI:** 10.1038/srep34544

**Published:** 2016-10-06

**Authors:** Silvia Bianchelli, Emanuela Buschi, Roberto Danovaro, Antonio Pusceddu

**Affiliations:** 1 Dipartimento di Scienze della Vita e dell’Ambiente, Università Politecnica delle Marche, Via Brecce Bianche, 60131 Ancona, Italy; 2 Stazione Zoologica Anton Dohrn, Villa Comunale 1, Napoli, Italy; 3 Dipartimento di Scienze della Vita e dell’Ambiente, Università degli Studi di Cagliari, Via Tommaso Fiorelli 1, 09126 Cagliari, Italy

## Abstract

In the Mediterranean Sea hard-bottom macroalgal meadows may switch to alternative and less-productive barrens grounds, as a result of sea urchins overgrazing. Meiofauna (and especially nematodes) represent key components of benthic ecosystems, are highly-diversified, sensitive to environmental change and anthropogenic impacts, but, so-far, have been neglected in studies on regime shifts. We report here that sedimentary organic matter contents, meiofaunal taxa richness and community composition, nematode α- and β-biodiversity vary significantly between alternative macroalgal and barren states. The observed differences are consistent in six areas spread across the Mediterranean Sea, irrespective of barren extent. Our results suggest also that the low biodiversity levels in barren states are the result of habitat loss/fragmentation, which is associated also with a lower availability of trophic resources. Furthermore, differences in meiofaunal and nematode abundance, biomass and diversity between macroalgal meadow and barren states persist when the latter is not fully formed, or consists of patches interspersed in macroalgal meadows. Since barren grounds are expanding rapidly along the Mediterranean Sea and meiofauna are a key trophic component in marine ecosystems, we suggest that the extension and persistence of barrens at the expenses of macroalgal meadows could also affect resilience of higher trophic level.

Sudden, hardly predictable and persistent shifts in the characteristics and functioning of ecosystems have been increasingly documented in terrestrial and marine environments worldwide^1^,^2^,^3^. In marine environments, regime shifts have been reported at all latitudes, from tropical to polar ecosystems (i.e., coral reefs, salt marshes, kelp forests, mangroves, Arctic and Antarctic sea ice). Shifts in either chemical and physical characteristics of marine ecosystems (i.e., thermohaline circulation, oxygen and substrate availability) can result in changes in biomass and biodiversity of communities, food web structure and ecosystem functions^3^. Regime shifts in marine environments are also related to reduced ecosystem resilience to disturbance, and, in turn, can cause the decline of the ecosystem services, ultimately impairing humans well-being^1^,^4^.

Studies conducted so far on benthic ecosystems showed that multiple stressors can drive and/or interact with regime shifts. Besides the effect of climate change (e.g., favouring as example the shift from corals to macroalgae dominance in tropical reefs^5^), direct anthropogenic pressures, including resource overexploitation, eutrophication and pollution, are known to play a prominent role in determining regime shifts at local/regional scales, which, in turn, foster consequences like habitat destruction and biodiversity loss^1^,^3^,^5^.

Regime shifts have also been observed in the Mediterranean Sea, a biodiversity hotspot threatened by several and often synergistic anthropogenic stressors^6^,^7^. Here, hard-bottom ecosystems dominated by macroalgal meadows (generally dominated by *Cystoseria ssp.*, one of the most important habitat-forming species of shallow Mediterranean Sea ecosystems^8^), may switch to alternative and less-productive systems defined as “barrens” where the macroalgal components are replaced by encrusting coralline algae^9^,^10^. This shift is caused by the rapid disappearance of macroalgal meadows, overgrazed by sea urchins. Such overgrazing is determined by sudden demographic explosions of sea urchins, favoured by the removal of their predators, typically fish of commercial interest^9^,^11^,^12^,^13^,^14^. Sea urchin overgrazing on macroalgae (as *Cystoseira spp.* in the Mediterranean Sea^8^) results in a loss of habitat complexity, a decrease of benthic and epiphytic macrofaunal biomass and biodiversity, and can decrease the resilience of degraded macroalgal meadows for decades^2^,^9^,^15^.

The diversified and productive habitats dominated by macroalgae and the impoverished barrens caused by sea urchin overgrazing are amongst the most distinctive alternative states of Mediterranean subtidal seascapes. Similar shifts have been described for kelp forests at different latitudes, where overgrazing by sea urchins has created barren grounds over thousands of kilometres worldwide (i.e., NE Pacific in the 1960–1970s, Norwegian coast in the 1970s, NW Atlantic in the 1970–1980s and Tasmania in the 2000s)^15^.

Wherever the alternative states have been formed, a recurrent impact reported after their establishment is the loss of biodiversity^3^. Most studies investigating biodiversity changes in alternative states in marine ecosystems have focused on macroalgae, macro- and megafauna biodiversity^9^,^11^,^12^,^13^,^14^,^15^,^16^, whereas the meiofauna have been, to the best of our knowledge, neglected, so far.

Meiofauna (and among them, nematodes) are the most abundant group of metazoans in the marine benthos. They are characterized by high levels of structural and functional biodiversity in all marine ecosystems, and in particular in the Mediterranean Sea^6^. Meiofauna play a key ecological role in linking detrital and prokaryotic components with higher trophic levels: in fact most of the meiofaunal taxa feed on microalgae, prokaryotes or detritus and, at the same time, are a food source for macro-, megafauna and fish^17^, thus representing a key trophic resource in marine food webs^18^. In addition, meiofauna and nematodes are known to respond rapidly to many different sources of natural and anthropogenic disturbance affecting benthic environments, from coastal habitats to the deep sea^19^,^20^,^21^,^22^,^23^,^24^. For these reasons, we contend that meiofauna and, particularly, nematodes may be affected by the habitat loss or fragmentation due to the replacement of *Cystoseira spp.* meadows with barren grounds. Moreover, since macroalgal meadows can provide higher resource availability to the benthos, we also affirm that the expected changes in meiofaunal attributes in barren grounds could be the result of changes in the availability of food resources.

To test the null hypothesis that meiofauna and nematode biodiversity did not vary between macroalgal meadow and barren states, we investigated meiofaunal abundance, biomass and diversity (in terms of richness of taxa and taxonomic composition), and nematode biodiversity (in terms of structural, functional/trophic biodiversity and assemblage composition) in six areas of the Western-Central Mediterranean Sea, characterized by the co-occurrence of either macroalgae meadows and barrens. In order to better understand how and how much and in which way meiofaunal biodiversity could be possibly affected by the formation of barren grounds, we also investigated the differences between barrens and meadows in terms of α-diversity (nematode species richness at each sampling point), habitat diversity (overall species richness in barrens and meadows) and β-diversity (nematode species composition, in terms of loss or turnover of species). Moreover, since changes in biodiversity among the states under scrutiny could be mediated by changes in the availability of resources, we also investigated differences in sedimentary organic matter (OM) quantity, biochemical composition and nutritional quality, as well as their putative role in driving meiofaunal and nematode diversity.

## Results

All the data dealing with the investigated variables are reported in Table 1a,b. The output of all statistical analyses is reported in the Supplementary Information (PERMDISP analyses on OM and meiofaunal variables in Supplementary Figs S1 and S2 respectively, PERMANOVA analyses on all variables at the different spatial scales Supplementary Tables S1 and S2).

### Sedimentary organic matter

The results of PERMANOVA on OM contents, biochemical composition and nutritional quality conducted at the largest spatial scale (i.e., among areas) revealed significant differences for all the investigated variables in both states, with only some exceptions (Supplementary Table S1a). The following pairwise tests revealed however that patterns in the observed differences in OM contents among areas varied in the two states, also depending on the variable considered (Table 1a).

The results of PERMANOVA conducted separately for each area revealed a significant effect of the factor State for almost all of the investigated variables (Supplementary Table S2a). More specifically, when significant differences were observed among states, the concentrations of all biochemical compounds and nutritional quality indicators were higher in meadows than in barrens at almost all areas and sites, with the exception of carbohydrate and biopolymeric C (Table 1a). The forest plots showed that barrens had significant negative effects on the concentration of all the investigated biochemical compounds, at almost all investigated areas (Supplementary Fig. S3).

The results of multivariate PERMANOVA conducted to ascertain variations in on the OM biochemical composition and the overall nutritional quality, separately for each area, revealed a significant effect of the factor State (i.e. barren vs. meadow) at all areas (Supplementary Table S2a).

### Meiofaunal abundance and biomass

The results of PERMANOVA on meiofaunal abundance and biomass at the largest spatial scale (among areas) revealed significant differences for both variables for either barrens or meadows (Supplementary Table S1b). Pairwise tests revealed however that patterns in the observed differences in meiofaunal abundance and biomass among areas varied in the two states.

**Figure 1:**
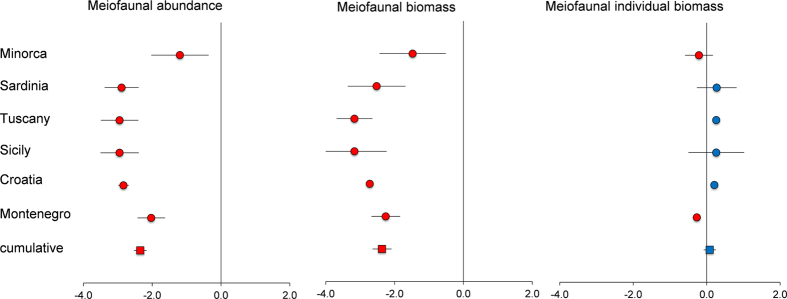
Forest plots showing the negative effect (red dots) determined by barrens on meiofaunal total abundance, total and individual biomass at all investigated areas (round symbol) and cumulatively for all areas (square symbol). Bars represent the standard error.

The results of univariate PERMANOVA revealed a significant effect of the factor State for meiofaunal abundance and biomass at all areas (Supplementary Table S2b). At all areas and sites meiofaunal abundance and biomass were greater in meadows than in barrens (Table 1b). The forest plots indicated a negative effect of the presence of barrens on both for meiofaunal total abundance and biomass, whereas a positive effect was observed on meiofaunal individual biomass (Fig. 1).

### Meiofaunal and nematode diversity

Overall, 24 meiofaunal higher taxa, of which 5 were exclusively found in meadows, and 174 nematode species, of which 12 and 78 were exclusively found in barrens and meadows respectively, were retrieved.

**Figure 2:**
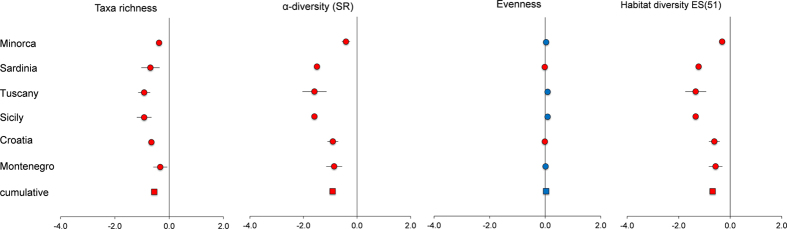
Forest plots showing the negative effect (red dots) determined by barrens on meiofaunal and nematode diversity at all investigated areas (round symbol) and cumulatively for all areas (square symbol). Bars represent the standard error.

The richness of meiofaunal higher taxa, nematode species richness (SR), Margalef index (D), Pielou evenness index (J), Shannon index (H′), expected species number (ES22 and ES51, for point and habitat diversity, respectively), index of trophic diversity (1-ITD), and maturity index (MI) are reported in Table 1b. Values of richness of meiofaunal taxa, nematode SR, ES(22), D, H′ and ES(51) were higher in meadows than in barrens consistently at all areas (Table 1b), whereas J, 1-ITD and MI were higher in meadows than in barrens only at some areas. The forest plots indicated a negative effect of the presence of barrens, on both meiofaunal and nematode diversity (both at point and habitat spatial scale) consistently in all areas, whereas a null effect was observed for the evenness (Fig. 2).

**Figure 3:**
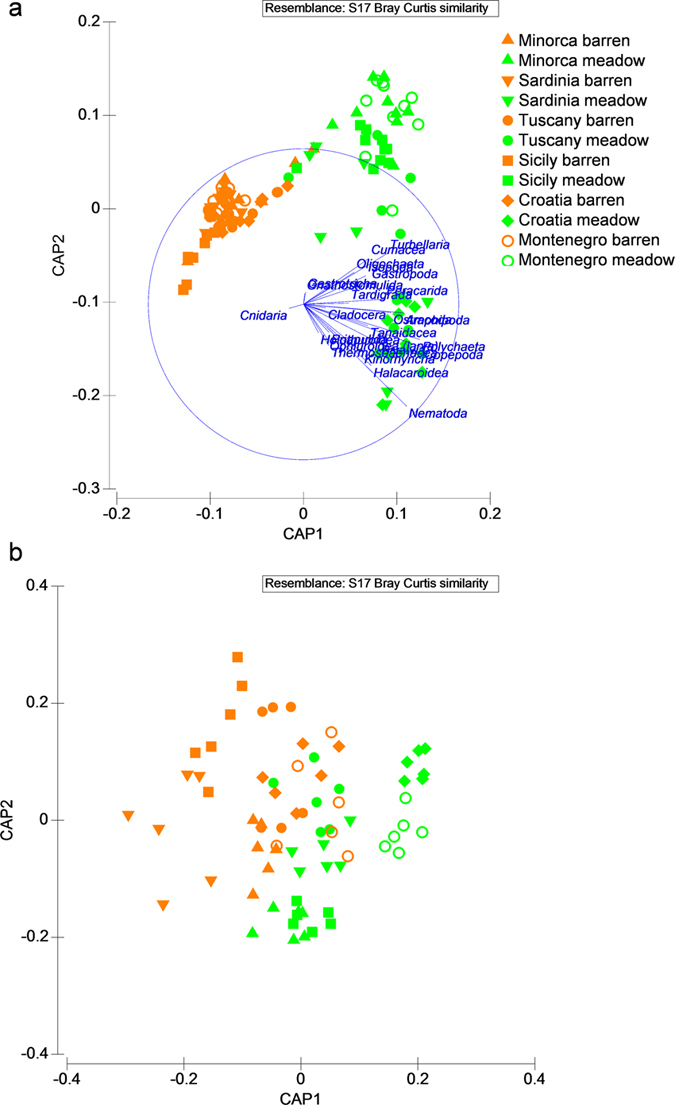
Output of CAP conducted on the meiofaunal community taxonomic composition (a) and on nematode species composition (b) In (**a**) vectors are proportional to the percentage of variance in the community composition explained by each meiofaunal taxon.

The results of the multivariate PERMANOVA carried out to ascertain variations in the composition of meiofaunal communities at the largest spatial scale (i.e., among areas) revealed significant variations either in barrens and meadows, but with different patterns for the two habitats (Supplementary Table S1b). The analysis conducted to ascertain differences in the composition of meiofaunal communities between barren and meadows, revealed a significant effect of the factor State at all areas (Supplementary Table S2b). The CAP analysis revealed at all areas clear segregations of meiofaunal communities hosted in barrens from those in meadows (Fig. 3a). Such segregations were driven by changes in the abundance of almost all of the meiofaunal taxa retrieved. In particular, Priapulida, Gnathostomulida, Gastrotricha, Holothuroidea and Thermosbanacea where exclusive of meadows at all areas, whereas Nematoda, Cumacea, and Peracarida were present exclusively or more abundant (depending on the area) in meadows than in barrens. Copepoda, Gastropoda, Halacaroidea and Cnidaria were consistently more abundant in barrens than in meadows. The dissimilarity in the taxonomic composition of meiofaunal communities between areas ranged from 32–58% and 31–57% for barrens and meadows, respectively (Table 2a). The dissimilarity between barrens and meadows communities at each area was greater than 75%, except that observed at Minorca (64%, Table 2b).

The results of the multivariate PERMANOVA carried out to assess variations in the species composition of nematode assemblages at the largest spatial scale (i.e., among areas) revealed significant differences either for barrens or meadows (Supplementary Table S1c). The pairwise tests revealed that significant differences occurred among all areas (with only one exception for barrens). The analysis conducted to ascertain, separately for each area, the effect of the factor State (i.e., barren vs. meadows) on the composition of nematode communities revealed significant differences only at one of the two sites in Sardinia, Sicily and Croatia and in Montenegro (Supplementary Table S2c). Nonetheless, the CAP revealed clear segregations between nematode assemblages inhabiting barrens and meadows sediments at all areas (Fig. 3b).

To ascertain whether and how the nematode β-diversity was driven by species turnover or species loss, we applied the Jaccard dissimilarity measures, at all the investigated spatial scales (Table 3). At the smallest (i.e., when comparing sites in each state and area) and largest (i.e., when comparing areas for each state separately) spatial scales the portion of β-diversity attributable to species turnover (β_JTU_) was much higher (up to more than one order of magnitude) than that attributable to species loss (β_JNE_). When comparing states in each area separately, β_JTU_ is 1.5–2.2 times higher than (β_JNE_) in Minorca and Croatia, whereas in all other areas β_JNE_ was higher (1.2–3 times) than β_JTU_.

At all areas and sites, nematode assemblages were dominated by epi-growth feeders (2A, feeding on microalgal biomass) in both states (on average, 59 and 65% in meadows and barrens, respectively), at all areas and sites, followed by predators/omnivores (2B; on average, 18 and 23% in meadows and barrens, respectively) and deposit feeders (cumulatively selective and non-selective, 1A + 1B; on average, 12 and 23% in barrens and meadows, respectively, Table 1b). The percentages of epi-growth feeders and predators/omnivores were higher in barrens than in meadows, whereas the percentage of deposit feeders was higher in meadows than in barrens.

### Relationships between trophic resources, meiofaunal abundance, biomass and biodiversity

The results of the DistLM forward analyses revealed that, at all areas, the variability in the meiofaunal abundance, biomass, richness of meiofaunal taxa and nematode species richness were mostly explained (up to more 90% of explained variance) by the % of barren coverage, followed in 3 of the 6 areas by the quantity of OM (in terms of biopolymeric C concentration, up to 50% of explained variance) (Supplementary Table S3). When considering the % of barren along with OM biochemical composition and nutritional quality, the observed variability in the meiofaunal abundance, biomass, richness of taxa and nematode SR were significantly explained by the % of barren (explaining up to 44% of variability), OM biochemical composition and nutritional quality (Supplementary Table S3).

## Discussion

### Loss of energy and meiofaunal standing stocks in barren systems

In the Mediterranean Sea, *Cystoseira* spp. are among the most important habitat-forming species of shallow ecosystems, and, as such, are responsible for the maintenance of abundant and biodiverse faunal and algal assemblages^8^. Thus, any loss in *Cystoseira* spp. coverage is conceivably associated with a reduction in benthic biomass and biodiversity. At the same time, it can be expected that biomass reduction associated with the loss of algal coverage could be also the result of a decrease in the quantity and nutritional quality of available resources^25^. This would hold specifically true for the meiofauna, whose abundance, biomass and biodiversity are tightly linked with the quantity and availability of resources^20^,^26^.

Accordingly, we show here that sedimentary organic matter contents were lower in barren systems than in meadows in all areas under scrutiny, for almost all the considered variables. Our results also show that the reduction in the amount of food for benthic consumers in barren grounds was also associated with a lower nutritional quality when compared with that in macroalgal meadows. These results indicate that the shift from macroalgal meadows to barren systems was associated with a considerable decrease in the quantity and nutritional quality of resources for benthic consumers. Our findings also highlight that meiofaunal abundance and biomass were lower in barrens than in macroalgal-dominated systems, consistently in all areas. Therefore, it can be argued that the reduction in meiofaunal standing stocks, previously reported also for other benthic components and oceanic regions^16^, could be the cumulative/synergistic consequence of the macroalgal meadows loss/fragmentation and of the decreased availability of trophic resources. Our results also indicate a positive effect of the presence of barrens on meiofaunal individual biomass. This, coupled to the significant decrease of meiofaunal biomass, indicates that the formation of barrens, associated to the loss of habitat complexity (provided by *Cystoseira spp.*), affected preferentially small individuals. This effect was also reported in previous studies investigating the impact of bottom trawling in deep-sea soft bottoms^21^.

### Consequences of the meadow-barren shift on meiofaunal α-biodiversity

Recent studies consistently reported that the loss of biodiversity is one of the major and most recurrent consequences of regime shifts and formation of alternative states, whatever the type of the shift and the affected ecosystem^4^.

We report here that at all areas the barren grounds were characterized by a reduced richness of meiofaunal higher taxa (26–47% loss on average). Priapulida, Gnathostomulida, Gastrotricha, Holothuroidea and Thermosbanacea, generally among the rarest taxa of meiofauna in shallow systems^20^, were absent in barren systems of almost all areas. This result suggests that the loss of habitat complexity, like the one offered by *Cystoseira spp.* canopy, can preferentially affect rare taxa, otherwise hosted in macroalgal meadows. On the other hand, Copepoda, Gastropoda, Halacaroidea and Cnidaria, being epi-benthic taxa and exploiting the availability of hard substrates more efficiently than burrowing taxa^28^, showed higher percentages in barrens than in meadows. These findings are consistent with results from previous manipulative studies conducted in Southern Australia, which demonstrated that the distribution of habitat-modifying species (i.e., the sea urchin *Centrostephanus rodgersii*, capable of forming barren grounds by overgrazing) can cause strong changes in the macro- and mega-benthic communities, leading to overwhelmingly impoverished communities, with a loss of 150 taxa in barren grounds typically associated with macroalgal meadows^16^. Overall, our results reflect the important role of the transition from macroalgal meadows to barren systems in deeply modifying the assets of meiofaunal biodiversity at the highest taxonomical levels.

Nematodes represent, generally, the most abundant taxon of meiofauna in most marine ecosystems, and are known to be a hyper-diverse taxon^29^. Indeed, changes in the biodiversity of this taxon are currently used as a descriptor of major consequences of a variety of natural and anthropogenic stressors on marine benthic ecosystems^21^,^23^. We show here that nematode species richness in barren systems was consistently lower (on average 25–57% loss) than in macroalgal meadows. This reduction is consistent with the decreased habitat quality because of the transition between macroalgal canopies and barren grounds, and indicates that the consequences of this typology of shift affected significantly also the smallest metazoan communities, otherwise supposed to be more resilient than larger organisms.

Overall our results indicate that the loss of *Cystoseira* spp. canopies observed in barren systems under scrutiny had severe consequences on stocks and biodiversity of meiofauna at different levels of taxonomical organization, from higher taxa to the species level. Since the loss of biodiversity in ecosystems characterised by the presence of habitat-forming species (e.g., coral reefs and seagrass meadows) can determine a decrease in rates of ecosystem functions^22^, we could hypothesize that the transition from macroalgal-dominated to barren grounds could have important consequences also on the overall function of the affected area. Nonetheless, we must acknowledge that changes in the strength and shape of the relationships between biodiversity and ecosystem functioning among alternative states deserve further investigations.

### Consequences of the meadow-barren shift on β-biodiversity and nematode functional traits

Theoretical ecology predicts that habitat heterogeneity allows a greater number of species to coexist, and, in turn, this would mean that any loss of habitat heterogeneity would result in a decrease of point (α) biodiversity. However, when habitat heterogeneity is fragmented or disrupted, as in the case of a shift from a macroalgal canopy to a barren ground, it can be envisaged that, along with a strong decrease in α-biodiversity (e.g. taxa or species richness), important changes in the composition of communities (i.e., β-diversity) would also occur.

As previously reported for other benthic components, such as macro- and megafaunal assemblages^16^, we report here that the decrease in the overall richness of meiofaunal taxa and nematode species in barren systems was also accompanied, at almost all areas, by changes in the composition of meiofaunal communities and nematode assemblages. These changes were partly due to the disappearance of certain taxa/species in barren grounds, but also to changes in their relative abundances in the two alternative states. At the higher taxonomic level, the compositional dissimilarity among meiofaunal communities inhabiting barrens and macroalgal meadows was very high at all areas, ranging from 64 to 88%, and, notably, such dissimilarity was most often greater than that observed among different areas within the same state.

At the species level, at all areas, nematode β-diversity (as Jaccard dissimilarity) between barrens and meadows was lower than that among areas for each of the two states separately. However, when the Jaccard dissimilarity was decomposed in its turnover and species loss components, it emerged that variations in the composition of nematode assemblages between meadows and barrens were due to loss of species more than to species turnover, at almost all areas. This result indicates that in both alternative states, spatial variability (i.e. among areas) in the composition of nematode assemblages were mostly explained by species replacement, which is consistent with the large heterogeneity of Mediterranean areas at the basin and sub-basin scale. On the other hand, irrespectively of the geographical area in which the barren has formed, the transition from algal canopies to barren grounds was mostly due to loss of biodiversity. This is also confirmed by the evenness and the maturity indices, which were high in both states in all investigated areas. This could suggest that the impact on abundance and species richness is non-selective, and that the species disappeared after ecosystem shift are not replaced by more opportunistic ones.

**Figure 4:**
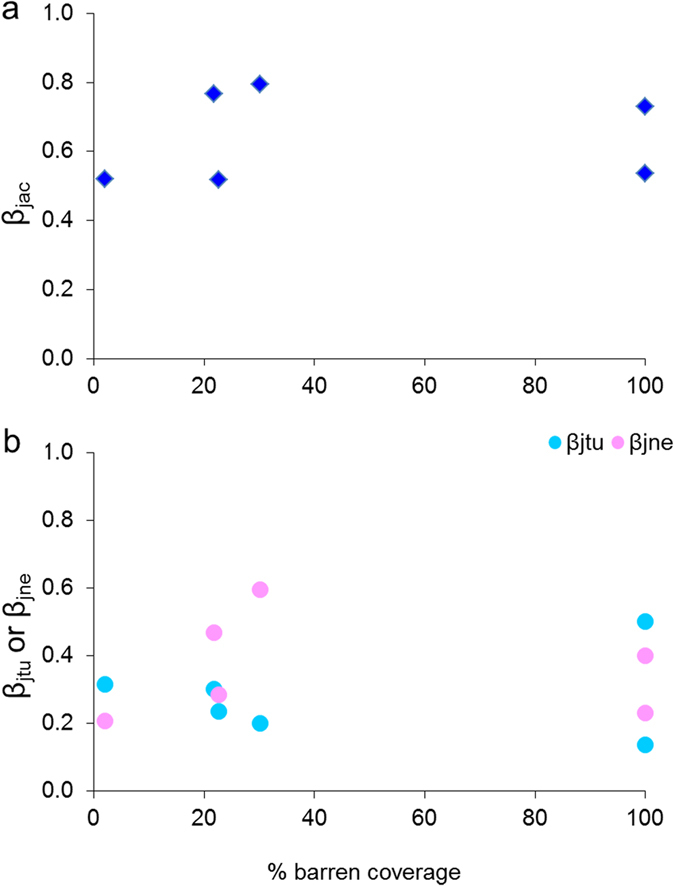
Relationships between % of barren coverage and β-diversity measures. (**a**) Jaccard β-diversity (β_jac_) and (**b**) turnover (β_jtu_) and loss of species (β_jne_) β-diversity components.

The transition to barrens usually does not hit on the entire meadow, but is often characterized by a patchy intermingled mosaic of the two states^30^,^31^,^32^. In this regard, previous studies have highlighted that the persistence of the barren is dependent upon the grazers (sea urchins), since they are incapable of maintaining barrens when their standing stocks fall below certain thresholds^15^. These findings would suggest that barrens of different sizes could lead to proportional loss of biodiversity. Counterintuitively, our results let us argue that the magnitude of biodiversity loss due to the regime shift was apparently independent from the extension of the barren grounds, either in hard bottoms fully covered by barrens or in those in which barrens are patchy. This argument is supported by the idiosyncratic relationship between the percentage of barren coverage and the values of Jaccard dissimilarity (and its turnover and species loss components) between the two alternative states (Fig. 4). Such a relationship indicated that changes of similar magnitude in the composition of nematode assemblages can be observed either in full or patchy barren grounds. This result suggests that, at least for the typology of shift under scrutiny, whatever the spatial extension of shift the consequences on (nematode) biodiversity were almost the same.

Nematodes include a variety of trophic guilds spanning from bacterivorous to deposit-feeder and predators. We found that the index of trophic diversity decreased significantly between meadows and barrens at two of the six investigated areas. This result suggests that the nematode species loss observed in the transition from macroagal meadows to barrens, according to our results, could determine a loss in functional biodiversity in 1/3 of the investigated systems.

The results of the present study clearly indicate strong differences in the availability of trophic resources and meiobenthic communities between the two alternative states, at both the higher meiofaunal taxa and nematode species level. Despite the differences observed in biodiversity among the areas in each of the two alternative states, which were due to the intrinsic and well-documented variability among Mediterranean sub-basins^6^, the observed differences between the alternative states were consistent, wherever the barren grounds have been formed.

Overall, our results show that meiofaunal assemblages responded to the shift, even when the barren was not yet fully formed, or consisted of small patches interspersed in algal meadows. Moreover, our results confirm that the presence of barrens were associated with a collapse of meiofaunal assemblages, conceivably with negative effects on the energy transfer to higher trophic levels^18^,^27^, thus impairing the provision of goods and services from these marine ecosystems.

## Methods

### Study area and data collection

**Figure 5:**
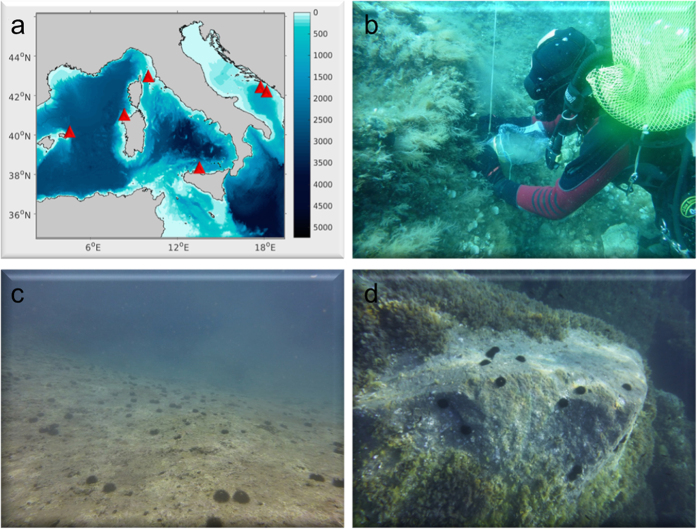
Sampling areas (**a**) and alternative states: meadows in Tuscany (**b**), full barren in Croatia, sampling Site 2 (**c**) and patchy barren in Sardinia (**d**). In (**a**) the map was generated using Matlab R2015b (8.6.0), Unix version 64-bit (glnxa64), www.mathworks.com, and modified using Microsoft Power Point (version 14.0.7166.5000, 32 bit). In (**b**) collection of samples is illustrated. The coordinates of sites in (**b**–**d**) are reported in Table 1.

Samples were collected June-September 2014 from six areas spread over a longitudinal gradient in the Western-Central Mediterranean Sea (Fig. 5, Table 1a): Minorca (Spain), Capraia (Tuscany, Italy), Sardinia (Italy), Ustica (Sicily, Italy) islands, Molunat (Croatia) and Tivat (Montenegro). All areas were characterized by the presence of erected macroalgae meadows (dominated by *Cystoseira* spp.) and barrens (dominated by encrusting coralline algae and sea urchins). At Molunat and Tivat, samples were collected from the two different ecosystems (meadows and barrens, at thousands of meters distance each other), at two different randomly selected sites (at hundreds meters distance each other). At Minorca, Sardinia, Tuscany and Sicily the barrens were formed by patches (at tens of meters distance each other) interspersed with the macroalgae meadows and samples were collected at two different randomly selected sites (at hundreds meters distance each other). At all sites, sampling was performed within the bathymetric range comprised between 4 and 6 m depth.

All samples were collected by SCUBA divers, using a modified manual corer enabled to scrape the hard bottom surface^28^. At each site, five replicate samples for meiofaunal analyses and three replicate samples for organic matter analyses were collected.

Samples for organic matter determinations were immediately frozen at −20 °C until analyses in the laboratory (within a few weeks), whereas samples for meiofauna and nematodes were immediately fixed with buffered formaldehyde (4% v/v final concentration in sodium tetraborate) and stained with 0.5 g L^−1^ Rose Bengal^33^.

### Biochemical composition of sedimentary organic matter

In the laboratory, the samples were analysed for organic matter (OM) biochemical composition in terms of phytopigment, protein, carbohydrate and lipid contents. Briefly, chlorophyll-a and phaeopigments were analysed fluorometrically. Total phytopigment concentrations, utilized as a proxy for the organic material of algal origin, were defined as the sum of chlorophyll-a and phaeopigment concentrations and converted into C equivalents^33^,^34^. Protein, carbohydrate and lipid analyses were carried out spectrophotometrically^33^,^34^. The concentrations were converted to C equivalents and their sum referred to biopolymeric C^33^,^34^. The percentage contribution of phytopigment and protein C to biopolymeric C contents and the values of protein to carbohydrate ratio were used as descriptors of nutritional quality of OM^35^.

### Meiofaunal abundance, biomass and diversity

Samples were sieved through a 1000-μm mesh, and a 20-μm mesh was used to retain the smallest organisms. The fraction remaining on the latter sieve was re-suspended and centrifuged three times with Ludox HS40 (final density of 1.18 g cm^−3^)^33^. All animals remaining in the surnatant were passed again through a 20 μm mesh net, washed with tap water and, after staining with Rose Bengal, sorted, counted and identified under a stereomicroscope (×40 magnification)^17^,^33^.

Meiofaunal biomass was assessed by bio-volumetric measurements for all specimens encountered. Nematode biomass was calculated from the biovolume (after being mounted on permanent slides), using the Andrassy’s^36^ formula (V = L × W^2^ × 0.063 × 10^−5^, in which body length L, and width W, are expressed in mm and volume V in nL). Body volumes of all other taxa were derived from measurements of body length and width (L and W in mm, respectively), using the formula V = L × W^2^ × C, where C is the approximate conversion factor for each meiofaunal taxon^37^. Each body volume was then multiplied by an average density (1.13 g cm^−3^) to obtain the biomass and the C content was considered to be 40% of the dry weight^37^,^38^. The meiofaunal individual biomass was obtained from the ratio between total abundance and biomass.

### Nematode biodiversity

From three of the five replicates of meiofaunal samples, 100 nematodes from each replicate (or all of the nematodes when the abundance was <100 specimens per sample) were mounted on permanent slides^33^. The nematodes were identified to the species level according to the presently used manuals^39^,^40^,^41^,^42^. All of the unknown species were indicated as sp_1_, sp_2_, sp_3_, … sp_n_.

The nematode α-diversity (i.e., point diversity^43^) was estimated using the species richness (SR). As species richness is strongly affected by sample size, the expected number of species (ES), which provides a standardisation of the values of the species richness according to the sample size, was also calculated. The ES for a theoretical sample of 22 (i.e., the minimum number of retrieved nematodes cumulatively for the three replicates, ES22) and 51 specimens (i.e, in meadows or barrens state, cumulatively for the sites for each state, ES51) was used. ES22 and ES51 were used to measure the sampling point and the habitat diversity, respectively. The point diversity was also measured by the Margalef (D), Shannon-Wiener (H′, using log-base 2) and evenness J indices^44^, calculated using PRIMER v6.0+^45^.

β-diversity of nematode assemblages between sites (for each state and area), states (for each area) and among areas (within each state) was estimated using the Jaccard’s dissimilarity and its turnover (β_jtu_; i.e., replacement of species) and nestedness (β_jne_; i.e., loss of species) components^46^,^47^.

Nematodes were divided into four groups, according to their individual stoma morphology^48^: no buccal cavity or a fine tubular selective (bacterial) feeders (1A); large but unarmed buccal cavity non-selective deposit feeders (1B); buccal cavity with scraping tooth or teeth, epistrate or epigrowth (diatoms) feeders (2A); and buccal cavity with large jaws, predators/omnivores (2B). The Index of Trophic Diversity (ITD) was calculated as 1-ITD, where ITD = g_1_^2^ + g_2_^2^ + g_3_^2^…+g_n_^2^ and g is the relative contribution of each trophic group to the total number of individuals and n is the number of trophic groups^33^.

To determine the life strategies of the nematodes, the maturity index (MI) was calculated according to the weighted mean of the individual genus scores, as 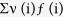, where ν is the colonisers-persisters (c-p) value of the genus i, and ƒ (i) is the frequency of that genus^49^.

### Statistical analyses

To assess differences between areas, states and sites, we applied uni- and multivariate distance-based permutational analyses of variance. All the statistical analyses were carried out using the same sampling design, considering 3 factors as main sources of variance: *State* (fixed, 2 levels: meadows and barrens), *Area* (fixed, 6 levels: Minorca, Sardinia, Tuscany, Sicily, Croatia and Montenegro) and *Site* (random and nested in *State* and *Area*, 2 levels: Site1 and Site2).

The variability in the OM compounds contents, total meiofaunal abundance and biomass were assessed using univariate distance-based permutational analyses of variance PERMANOVA^50^,^51^, separately for each variable.

The variability in the biochemical composition and nutritional quality of OM, taxonomic/species composition of meiofaunal communities and nematode assemblages were assessed using multivariate distance-based permutational analyses of variance, PERMANOVA^50^,^51^. We used the PERMANOVA tests based on matrices of Euclidean distance after normalisation of the data (for OM variables) and on Bray-Curtis similarity resemblance matrix without transformation (for meiofaunal abundance, biomass and nematode species composition)^50^,^51^.

Since PERMANOVA is sensitive to differences in multivariate dispersion among groups, we also used a test of homogeneity of dispersion (PERMDISP) to test the null hypothesis of equal dispersions among groups as an analogous to a uni-variate test for homogeneity prior to identifying differences in the distribution among groups at the different spatial scales. When significant differences were encountered, the PERMANOVA analyses were conducted separately for each area or sampling site. However, in order to gather additional information on the variability of investigated variables at largest spatial scale (i.e., among areas), we forced the PERMANOVA analysis, testing for differences among areas, for barrens and meadows separately.

To visualize differences between states, areas and sites in the meiofaunal community and nematode assemblages’ composition, bi-plots after a Canonical Analysis of Principal Coordinates (CAP) were prepared^52^. Additionally, in order to quantify the % dissimilarity among areas within each state and between states at each area, SIMPER tests were also carried out.

For all the statistical analyses utilized, when significant differences were observed between states, areas or sites, pairwise tests were also applied to ascertain patterns of differences among samples (pairwise results shown only for differences among areas).

To determine whether the indices of diversity (in terms of richness of meiofaunal taxa and nematode SR) are influenced by the environmental variables (% of barren, quantity of sedimentary OM in terms of biopolymeric C concentration and OM biochemical composition), we carried out multivariate multiple regression analyses (DistLM forward). P values were obtained with 4999 permutations of residuals under the reduced model. Linear relationships between quantity of sediment organic matter and between the meiofaunal and nematode diversity indices were also investigated using linear regressions, in order to evaluate the direction and scale of variations. Uni- and multivariate PERMANOVA, pairwise, CAP, SIMPER and DistLM forward tests were carried out using the routines included in the software PRIMER 6+^53^.

To visualize the differences between meadows and barrens for all the investigated variables we first estimated the effect sizes with log–response ratios^54^,^55^: R_i_ = ln (X_Bi_/X_Mi_), where R_i_ is the log–response ratio for the response category (i.e., barren conditions) of the area i, and X_Bi_ and X_Mi_ are the mean values of the metric for area i in barrens (B) and meadows (M), respectively.

## Additional Information

**How to cite this article**: Bianchelli, S. *et al*. Biodiversity loss and turnover in alternative states in the Mediterranean Sea: a case study on meiofauna. *Sci. Rep.* **6**, 34544; doi: 10.1038/srep34544 (2016).

## Supplementary Material

Supplementary Information

## Figures and Tables

**Table 1:** Organic matter biochemical compounds contents, indicators of nutritional quality (a) and total meiofaunal abundance and biomass, richness of taxa, nematode diversity indices (SR, D, J, H′(log_e_), ES(22), ES(51), 1-ITD and MI) and trophic structure (b).

(a)			Latitude	Longitude	Total phytopigment		Protein		Carbohydrate		Lipid		Biopolymeric C		Phytopigment: Biopolymeric C	Protein: Biopolymeric C	Protein: Carbohydrate		
					(μg g^−1^)		(mg g^−1^)		(mg g^−1^)		(mg g^−1^)		(mg g^−1^)							
			(°N)	(°E)	avg	sd	avg	sd	avg	sd	avg	sd	avg	sd	(%)	(%)				
Minorca	Barren	Site1	40°03′37″	03°59′34″	523.8	116.3	10.2	2.0	18.1	1.9	0.6	0.0	12.7	1.8	44.2	39.5	0.6		
		Site2	40°03′37″	03°59′34″	69.3	14.9	2.1	0.2	3.5	0.6	0.3	0.0	2.7	0.2	27.9	37.8	0.6		
	Meadow	Site1	40°03′39″	04°00′11″	3401.6	555.8	6.3	0.3	34.4	5.5	3.3	0.3	19.4	2.3	na	16.0	0.2		
		Site2	40°03′39″	04°00′11″	2300.0	892.9	3.0	0.5	6.2	0.4	0.4	0.0	4.3	0.4	na	34.6	0.5		
Sardinia	Barren	Site1	40°58′16″	08°11′46″	251.6	11.2	4.4	0.4	35.3	3.2	2.8	0.3	18.4	1.7	10.7	11.7	0.1		
		Site2	40°56′19′	08°10′35″	157.6	30.8	2.2	0.3	9.0	1.4	4.7	0.5	8.3	0.4	18.2	13.3	0.2		
	Meadow	Site1	40°58′16″	08°11′46″	380.0	0.5	12.9	0.8	15.0	2.0	2.8	0.5	14.4	0.8	32.6	43.8	0.9		
		Site2	40°56′19′	08°10′35″	260.5	28.8	13.3	1.8	5.8	1.6	2.6	0.1	10.8	1.5	26.3	60.6	2.3		
Tuscany	Barren	Site1	43°01′05″	09°49′39″	103.4	34.5	2.6	0.1	24.8	0.6	0.3	0.0	11.5	0.3	13.9	11.2	0.1		
		Site2	43°01′05″	09°49′39″	254.1	8.9	4.3	0.4	12.4	0.8	1.8	0.3	8.4	0.3	45.8	25.2	0.3		
	Meadow	Site1	43°00′23″	09°49′03″	801.5	448.6	12.9	1.6	11.2	0.3	1.3	0.3	11.8	0.9	96.2	53.8	1.2		
		Site2	43°00′23″	09°49′03″	618.5	91.9	5.6	0.2	7.3	2.0	2.5	0.3	7.5	1.0	na	36.7	0.8		
Sicily	Barren	Site1	38°41′57″	13°11′13″	99.1	3.0	4.2	0.4	9.5	1.0	3.3	0.2	8.3	0.2	9.3	24.9	0.4		
		Site2	38°42′14″	13°11′40″	35.0	1.0	3.4	0.2	1.1	0.1	0.8	0.1	2.7	0.2	12.7	61.6	3.2		
	Meadow	Site1	38°41′57″	13°11′13″	257.5	15.5	23.6	2.8	20.5	1.6	5.4	0.2	23.8	0.7	5.8	48.5	1.2		
		Site2	38°42′14″	13°11′40″	168.8	15.8	13.4	2.9	9.6	0.4	1.1	0.0	11.2	1.3	15.3	58.5	1.4		
Croatia	Barren	Site1	42°28′3″	18°24′39″	538.6	81.1	7.2	0.7	57.4	16.6	2.9	0.3	28.7	6.5	24.0	12.2	0.1		
		Site2	42°26′28″	18°25′55″	267.2	106.6	4.5	0.8	28.1	3.9	0.6	0.1	13.9	2.0	23.3	15.9	0.2		
	Meadow	Site1	42°27′47″	18°25′16″	279.4	132.1	10.0	1.0	16.3	1.9	1.4	0.1	12.5	1.2	7.4	39.4	0.6		
		Site2	42°27′36″	18°24′37″	299.2	17.2	8.3	1.5	14.3	3.5	3.1	0.2	12.2	2.3	11.8	33.5	0.6		
Montenegro	Barren	Site1	42°21′48″	18°37′59″	311.4	42.9	6.7	0.1	46.5	3.6	1.4	0.2	22.9	1.2	7.9	14.3	0.1		
		Site2	42°23′42″	18°33′38″	374.4	69.6	1.8	0.3	30.8	4.8	2.8	0.3	15.3	2.2	6.6	5.7	0.1		
	Meadow	Site1	42°21′48″	18°37′59″	166.3	105.7	12.1	2.5	10.4	1.9	1.9	0.3	11.5	1.2	13.4	51.5	1.2		
		Site2	42°23′47″	18°33′33″	90.7	15.8	4.7	0.3	8.8	0.4	3.4	0.0	8.4	0.0	6.0	27.7	0.5		
			**Total meiofaunal abundance (ind 10 cm**^**−2**^)		**Total meiofaunal biomass (μgC 10 cm**^**−2**^)		**Richness of taxa**	**Nematode diversity indeces**								**Nematode trophic groups**			
**(b)**			**avg**	**sd**	**avg**	**sd**	**N. taxa tot**	**SR**	**d**	**J**	**H′(loge)**	**ES(22)**	**ES(51)**	**1-ITD**	**MI**	**1A+1B%**	**2A%**	**2B%**	
Minorca	Barrens	Site1	57.7	32.7	11.7	4.3	10.0	34	5.8	0.8	2.7	10.9	16.8	0.3	2.7	5.1	79.3	15.6	
		Site2	24.9	11.5	4.5	1.1	9.0	30	6.0	0.8	2.8	12.9	19.8	0.4	3.0	4.0	73.6	22.4	
	Meadows	Site1	182.7	154.8	31.7	9.5	14.0	47	8.3	0.8	3.0	12.3	22.2	0.3	3.0	11.0	82.7	6.3	
		Site2	213.7	61.3	43.6	8.7	14.0	49	8.4	0.8	3.0	12.1	20.4	0.3	3.1	9.3	83.0	7.7	
Sardinia	Barrens	Site1	21.4	14.5	7.2	2.9	12.0	12	2.5	0.8	2.1	8.6	11.0	0.2	2.6	1.2	88.9	9.9	
		Site2	12.6	9.7	3.6	1.3	8.0	14	3.7	0.9	2.3	14.6	na	0.2	2.6	2.9	88.6	8.6	
	Meadows	Site1	397.9	171.4	225.8	97.5	17.0	47	8.1	0.9	3.3	10.7	23.7	0.6	3.0	20.0	61.7	18.3	
		Site2	172.2	103.8	44.9	14.2	18.0	57	9.8	0.9	3.4	15.3	25.3	0.6	3.0	21.3	60.3	18.3	
Tuscany	Barrens	Site1	39.2	19.4	9.3	2.4	15.0	37	7.5	0.9	3.3	15.4	25.3	0.7	3.2	28.6	42.0	29.4	
		Site2	38.4	23.8	8.3	2.0	12.0	20	5.2	0.9	2.8	14.6	na	0.6	3.1	20.0	52.5	27.5	
	Meadows	Site1	235.9	122.8	49.9	12.1	17.0	50	8.7	0.9	3.3	13.9	24.6	0.6	3.1	36.9	46.7	16.4	
		Site2	465.4	166.1	96.0	19.5	21.0	64	11.1	0.8	3.5	14.8	26.3	0.6	3.1	30.7	48.4	20.9	
Sicily	Barrens	Site1	16.0	14.0	2.8	1.6	8.0	11	3.2	0.9	2.2	11.0	na	0.6	3.3	18.2	36.4	45.5	
		Site2	13.3	11.8	2.2	0.7	10.0	12	3.3	1.0	2.4	13.9	na	0.5	2.9	3.6	67.9	28.6	
	Meadows	Site1	225.3	77.7	36.0	9.4	16.0	41	7.0	0.9	3.2	11.2	21.7	0.5	3.1	12.3	64.0	23.7	
		Site2	204.0	79.1	35.0	7.0	18.0	38	6.5	0.9	3.1	13.7	21.4	0.5	3.1	7.0	64.7	28.3	
Croatia	Barrens	Site1	32.8	14.5	9.8	2.3	11.0	21	4.1	0.7	2.3	9.3	14.1	0.3	2.8	9.6	81.6	8.8	
		Site2	51.0	22.1	7.6	0.7	10.0	31	6.0	0.9	3.1	16.2	22.0	0.5	2.7	16.7	66.7	16.7	
	Meadows	Site1	669.7	82.6	130.6	15.8	17.0	68	11.7	0.9	3.7	14.0	28.8	0.7	3.1	35.0	51.2	13.9	
		Site2	630.7	242.1	120.3	22.2	18.0	55	9.4	0.8	3.3	14.5	24.3	0.7	3.0	34.1	47.9	18.0	
Montenegro	Barrens	Site1	31.3	14.3	5.2	1.4	12.0	23	5.3	0.9	2.8	13.4	20.8	0.6	3.2	17.2	54.7	28.1	
		Site2	30.9	6.1	6.9	0.8	11.0	33	6.5	0.9	3.0	16.1	21.4	0.6	3.2	15.4	52.9	31.6	
	Meadows	Site1	290.3	156.1	68.5	17.7	16.0	62	10.7	0.9	3.6	13.3	27.8	0.7	3.3	30.0	45.3	24.7	
		Site2	183.4	35.7	58.6	14.0	15.0	59	10.2	0.9	3.6	16.2	27.7	0.6	2.9	28.0	53.3	18.7	

na = not available.

**Table 2:** Output of SIMPER analysis to assess the % dissimilarity in the meiofaunal communities among areas within barrens and meadows (a) and between alternative states at each investigated area (b).

(a)			SIMPER	
			% Dissimilarity within barrens	% Dissimilarity within meadows
	Among areas	Minorca vs Sardinia	50.6	50.73
		Minorca vs Tuscany	38.8	42.94
		Minorca vs Sicily	58.1	33.32
		Minorca vs Croatia	37.9	57.34
		Minorca vs Montenegro	36.8	32.22
		Sardinia vs Tuscany	48.9	44.22
		Sardinia vs Siciliy	47.1	51.98
		Sardinia vs Croatia	50.7	47.94
		Sardinia vs Montenegro	45.1	44.11
		Tuscany vs Sicily	55.3	37.62
		Tuscany vs Croatia	33.6	38.07
		Tuscany vs Montenegro	31.7	37.81
		Sicily vs Croatia	56.5	51.7
		Siciliy vs Montenegro	50.5	31.34
		Croatia vs Montenegro	31.7	52.24
**(b)**			**SIMPER**	
			**% Dissimilarity**	**Taxa responsible**
	Barren vs meadow	Minorca	63.8	Copepoda, Nematoda, Poychaeta
		Sardinia	85.8	Nematoda, Copepoda, Polychaeta
		Tuscany	75.3	Copepoda, Nematoda
		Sicily	86.5	Copepoda, Nematoda
		Croatia	87.5	Nematoda, Copepoda
		Montenegro	74.8	Copepoda, Nematoda, Poychaeta

**Table 3:** Total (β_jac_), turnover (β_jtu_), and nestedness (β_jne_) components of Jaccard dissimilarity in nematode assemblages: (a) among sites in each area and state, (b) between states in each area, and (c) among areas in each state.

(a)	Contrast	Minorca	barren	β_jac_	β_jtu_	β_jne_
	Site 1 vs Site 2			0.51	0.46	0.05
			meadows	0.52	0.51	0.02
		Sardinia	barren	0.56	0.50	0.06
			meadows	0.63	0.58	0.06
		Tuscany	barren	0.73	0.57	0.16
			meadows	0.48	0.36	0.12
		Sicily	barren	0.65	0.63	0.02
			meadows	0.63	0.62	0.01
		Croatia	barren	0.67	0.55	0.11
			meadows	0.52	0.41	0.11
		Montenegro	barren	0.63	0.52	0.12
			meadows	0.47	0.45	0.02
(b)	Barren vs Meadow	Minorca		0.52	0.31	0.21
		Sardinia		0.79	0.20	0.59
		Tuscany		0.52	0.24	0.28
		Sicily		0.77	0.30	0.47
		Croatia		0.73	0.50	0.23
		Montenegro		0.54	0.14	0.40
(c)	Among areas		barren	0.84	0.77	0.07
			meadows	0.78	0.74	0.04
