# Supplementary material for: Biodiversity loss and turnover in alternative states in the Mediterranean Sea: a case study on meiofauna
Source: Sci Rep. 2016 Oct 6;6:34544. doi: 10.1038/srep34544 (PMC5052579; doi:10.1038/srep34544)

Biodiversity loss and turnover in alternative states in the Mediterranean Sea: a case study on meiofauna

Silvia Bianchelli*, Emanuela Buschi, Roberto Danovaro, Antonio Pusceddu

*Corresponding author: silvia.bianchelli@univpm.it

LIST OF SUPPLEMENTARY INFORMATION

Supplementary Table S1. PERMANOVA and pairwise output to test differences among areas in OM, meiofaunal and nematode variables.

Supplementary Table S2. Results of PERMANOVA main test to test differences between alternative states and sites in OM, meiofaunal and nematode variables.

Supplementary Table S3. Results of DistLM forward carried out to ascertain the role of different environmental variables (OM quantity, biochemical composition and nutritional quality) on meiofaunal abundance, biomass, richness of taxa and nematode species richness. F=F statistic; *** = $P < 0.001$; ** = $P < 0.01$; * = $P < 0.05$; ns = not significant.

Supplementary Figure S1. Output of PERMDISP on OM variables.

Supplementary Figure S2. Output of PERMDISP on meiofaunal and nematode variables.

Supplementary Figure S3. Forest plots showing the negative effect (red dots) of barrens on OM biochemical compounds concentration at all investigated areas (round symbol) and cumulatively for all areas (square symbol). Bars represent the standard error.

Table S1. PERMANOVA and pairwise output to test differences among Areas in OM biochemical compounds contents and biochemical composition (a), meiofaunal abundance, biomass and taxonomic composition (b) and nematode species composition (c). df=degree of freedom, MS=means of squares; F= variance ratio, P= significance level, ns=not significant, *=P<0.05, **=P<0.01, ***=P<0.001, Min=Minorca, Sar=Sardinia, Tus=Tuscany, Sic=Sicily, Cro=Croatia and Mon=Montenegro.

			Source	df	MS	F	P	Pairwise
a)	Total phytopigment	Barren	Area	5	3.1	4.8	**	Cro>Mon>Min,Sar,Tus>Sic
			Residual	30	0.6			
		Meadow	Area	5	6.1	42.9	***	Min>Sar,Tus>Sic,Cro>Mon
			Residual	30	0.1			
	Protein	Barren	Area	5	1.4	1.6	ns	ns
			Residual	30	0.9			
		Meadow	Area	5	4.4	10.0	***	Sic>Sar>Tus,Cro,Mon>Min
			Residual	30	0.4			
	Carbohydrate	Barren	Area	5	4.4	9.9	***	Cro,Mon>Sar,Tus,Min,Sic
			Residual	30	0.4			
		Meadow	Area	5	1.8	2.1	ns	ns
			Residual	30	0.9			
	Lipid	Barren	Area	5	3.9	7.5	***	Sar>Tus,Sic,Cro,Mon>Min
			Residual	30	0.5			
		Meadow	Area	5	1.0	1.0	ns	ns
			Residual	30	1.0			
	Biopolymeric C	Barren	Area	5	4.0	8.0	***	Cro,Mon>Sar,Tus>Min,Sic
			Residual	30	0.5			
		Meadow	Area	5	1.8	2.1	ns	ns
			Residual	30	0.9			
	Biochemical composition	Barren	Area	5	16.6	5.4	***	Min≠Sar≠Tus≠Sic≠Cro≠Mon
			Residual	30	3.1			
		Meadow	Area	5	19.5	7.5	***	Min≠Sar,Sic≠Tus≠Cro,Mon
			Residual	30	2.6			
	Nutritional quality	Barren	Area	5	2011.4	11.0	***	Min≠Sar,Tus≠Sic≠Cro≠Mon
			Residual	30	182.1			
		Meadow	Area	5	1885.0	3.8	**	Min,Tus≠Sar≠Sic≠Cro,Mon
			Residual	30	491.7			
b)	Meiofaunal abundance	Barren	Area	5	3837.5	5.0	***	Min,Tus,Cro,Mon>Sar>Sic
			Residual	54	762.9			
		Meadow	Area	5	3580.1	6.3	***	Cro>Min,Sar,Tus,Sic,Mon
			Residual	54	572.7			
	Meiofaunal biomass	Barren	Area	5	3756.2	3.8	***	Cro,Min,Tus>Sar,Mon>Sic
			Residual	54	992.4			
		Meadow	Area	5	3758.4	4.6	***	Sar,Cro>Tus,Mon>Min,Sic
			Residual	54	811.7			
	Meiofaunal taxonomic composition	Barren	Area	5	4080.0	4.5	***	Min≠Sar,Sic≠Tus,Cro,Mon
			Residual	54	910.4			
		Meadow	Area	5	5097.2	7.4	***	Min,Tus,Cro,Mon≠Sar,Sic
			Residual	54	686.0			
c)	Nematode species composition	Barren	Area	5	7924.0	3.0	***	Min≠Sar≠Sic≠Cro≠Tus,Mon
			Residual	54	2616.0			
		Meadow	Area	5	7973.4	5.2	***	Min≠Sar≠Tus≠Sic≠Cro≠Mon
			Residual	54	1527.4			

Table S2. Results of PERMANOVA main test to test differences between alternative states and sites on OM biochemical compounds contents and biochemical composition (a), meiofaunal abundance, biomass and taxonomic composition (b) and nematode species composition (c). F= variance ratio, P= significance level, %var=% of explained variance, ns=not significant, *=P<0.05, **=P<0.01, ***=P<0.001; S1=Site1, S2=Site2).

	Minorca					Sardinia				
		Source	F	P	%var		Source	F	P	%var
a) Total phytopigment		State	18.4	*	85.1		State	2.3	ns	38.4
		Site(State)	3.8	ns	7.2		Site(State)	36.5	***	56.8
		Residual			7.7		Residual			4.8
Protein	S1	State	11.1	*	77.0		State	79.3	*	96.2
		Residual			23.0		Site(State)	3.6	ns	1.8
	S2	State	11.9	*	78.3		Residual			2.0
		Residual			21.7					
Carbohydrate	S1	State	23.5	**	88.2	S1	State	87.2	**	96.6
		Residual			11.8		Residual			3.4
	S2	State	40.7	**	93.0	S2	State	6.7	ns	65.7
		Residual			7.0		Residual			34.3
Lipid	S1	State	312.4	***	99.0	S1	State	0.0	ns	0.0
		Residual			1.0		Residual			100.0
	S2	State	191.5	***	98.4	S2	State	49.2	**	94.1
		Residual			1.6		Residual			5.9
Biopolymeric C	S1	State	15.8	*	83.2	S1	State	13.8	*	81.0
		Residual			16.8		Residual			19.0
	S2	State	39.7	**	92.8	S2	State	8.3	*	70.8
		Residual			7.2		Residual			29.2
Biochemical composition	S1	State	33.4	**	91.5	S1	State	12.7	**	79.6
		Residual			8.5		Residual			20.4
	S2	State	25.8	**	89.2	S2	State	19.7	**	86.2
		Residual			10.8		Residual			13.8
Nutritional quality		State	10.0	*	76.3	S1	State	273.	***	98.9
		Site(State)	5.0	*	13.6		Residual			1.1
		Residual			10.1	S2	State	1217	***	99.8
							Residual			0.2
b) Meiofaunal abundance		State	8.7	*	56.2		State	13.6	*	72.8
		Site(State)	2.0	ns	7.3		Site(State)	3.0	*	7.6
		Residual			36.4		Residual			19.6
Meiofaunal biomass		State	10.6	*	51.1		State	6.7	*	54.9
		Site(State)	1.1	ns	1.0		Site(State)	3.0	*	12.7
		Residual			47.9		Residual			32.4
Meiofaunal taxonomic composition		State	9.0	*	52.0		State	14.1	**	69.0
		Site(State)	1.5	ns	4.3		Site(State)	2.1	ns	5.4
		Residual			43.7		Residual			25.6
c) Nematode species composition		State	1.5	ns	11.9	S1	State	2.9	ns	38.9
		Site(State)	1.9	ns	20.9		Residual			61.1
		Residual			67.2	S2	State	3.6	*	46.7
							Residual			53.3

	Tuscany					Sicily				
		Source	F	P	%var		Source	F	P	%var
a) Total phytopigment		State	20.1	ns	71.8	S1	State	300.3	***	99.0
		Site(State)	0.8	ns	0.0		Residual			1.0
		Residual			28.2	S2	State	213.2	***	98.6
							Residual			1.4
Protein	S1	State	119.0	**	97.5	S1	State	138.3	***	97.9
		Residual			2.5		Residual			2.1
	S2	State	30.0	**	90.6	S2	State	35.1	**	91.9
		Residual			9.4		Residual			8.1
Carbohydrate	S1	State	1275.	***	99.8	S1	State	104.0	**	97.2
		Residual			0.2		Residual			2.8
	S2	State	17.8	*	84.9	S2	State	1636.	***	99.8
		Residual			15.1		Residual			0.2
Lipid		State	0.8	ns	0.0	S1	State	123.4	***	97.6
		Site(State)	43.2	***	93.4		Residual			2.4
		Residual			6.6	S2	State	18.3	*	85.2
							Residual			14.8
Biopolymeric C		State	0.0	ns	0.0	S1	State	1484.	***	99.8
		Site(State)	43.5	**	93.4		Residual			0.2
		Residual			6.6	S2	State	126.0	**	97.7
							Residual			2.3
Biochemical composition		State	2.846	ns	43.0	S1	State	125.6	***	97.6
		Site(State)	8.858	***	41.3		Residual			2.4
		Residual			15.8	S2	State	50.4	**	94.3
							Residual			5.7
Nutritional quality	S1	State	15.9	ns	83.2	S1	State	61.8	**	95.3
		Residual			16.8		Residual			4.7
	S2	State	0.5	ns	0.0	S2	State	1.3	ns	10.0
		Residual			100.0		Residual			90.0
b) Meiofaunal abundance		State	20.9	**	74.8		State	173.9	***	50.0
		Site(State)	1.7	ns	3.1		Site(State	0.2	ns	0.0
		Residual			22.1		Residual			50.0
Meiofaunal biomass		State	23.6	**	74.6		State	63.2	***	67.0
		Site(State)	1.4	ns	1.9		Site(State	0.3	ns	0.0
		Residual			23.6		Residual			33.0
Meiofaunal taxonomic composition		State	16.0	**	70.4		State	68.8	**	74.5
		Site(State)	1.9	ns	4.4		Site(State	0.4	ns	0.0
		Residual			25.2		Residual			25.5
c) Nematode species composition	S1	State	1.3	ns	7.9	S1	State	7.0	*	66.6
		Residual			92.1		Residual			33.4
	S2	State	2.5	ns	33.6	S2	State	2.9	ns	39.2
		Residual			66.4		Residual			60.8

	Croatia					Montenegro				
		Source	F	P	%var		Source	F	P	%var
a) Total phytopigment		State	0.7	ns	0.0		State	19.0	*	80.0
		Site(State)	6.2	*	63.5		Site(State)	1.6	ns	3.4
		Residual			36.5		Residual			16.6
Protein		State	4.5	ns	57.2		State	0.9	ns	0.0
		Site(State)	6.6	*	27.8		Site(State)	35.1	***	91.9
		Residual			14.9		Residual			8.1
Carbohydrate	S1	State	18.1	*	85.1	S1	State	234.	***	98.7
		Residual			14.9		Residual			1.3
	S2	State	20.7	*	86.8	S2	State	61.2	**	95.3
		Residual			13.2		Residual			4.7
Lipid	S1	State	66.9	**	95.6		State	0.3	ns	0.0
		Residual			4.4		Site(State)	59.7	***	95.1
	S2	State	400.1	***	99.3		Residual			4.9
		Residual			0.7					
Biopolymeric C	S1	State	17.7	*	84.8	S1	State	129.	***	97.7
		Residual			15.2		Residual			2.3
	S2	State	1.0	ns	0.0	S2	State	30.2	**	90.7
		Residual			100.0		Residual			9.3
Biochemical composition	S1	State	13.3	**	80.4		State	2.0	ns	29.6
		Residual			19.6		Site(State)	10.6	***	53.7
	S2	State	12.3	**	79.0		Residual			16.7
		Residual			21.0					
Nutritional quality		State	42.0	**	91.7	S1	Ha	26.7	**	89.6
		Site(State)	2.4	ns	2.6		Res			10.4
		Residual			5.7	S2	Ha	95.7	**	96.9
							Res			3.1
b) Meiofaunal abundance		State	69.4	***	90.1		State	63.7	**	88.1
		Site(State)	1.4	ns	0.8		Site(State)	1.2	ns	0.5
		Residual			9.1		Residual			11.4
Meiofaunal biomass		State	207.4	***	91.0		State	52.2	***	81.0
		Site(State)	0.5	ns	0.0		Site(State)	0.8	ns	0.0
		Residual			9.0		Residual			19.0
Meiofaunal taxonomic composition		State	36.1	**	86.2		State	56.6	***	85.3
		Site(State)	2.2	ns	2.7		Site(State)	1.1	ns	0.2
		Residual			11.2		Residual			14.5
c) Nematode species composition	S1	State	4.3	*	52.1		State	2.7	*	25.2
		Residual			47.9		Site(State)	1.3	ns	7.0
	S2	State	2.7	ns	36.0		Residual			67.9
		Residual			64.0					

Table S3. Results of DistLM forward carried out to ascertain the role of different environmental variables (OM quantity, biochemical composition and nutritional quality) on the observed variability in meiofaunal abundance, biomass, richness of taxa and nematode species richness. F=F statistic; *** = P < 0.001; ** = P < 0.01; * = P < 0.05; ns = not significant.

			Meiofaunal abundance			Meiofaunal biomass			Richness of meiofaunal taxa			Nematode Species Richness		
		Variable	F	P	%Explained variance	F	P	%Explained variance	F	P	%Explained variance	F	P	%Explained variance
A)	Minorca	% barren	4.4	*	86.6	3.0	ns	83.5	6.8	*	97.8	9.5	*	73.0
		biopolymeric C	0.5	ns	13.4	0.5	ns	16.5	0.1	ns	2.2	2.2	ns	27.0
	Sardinia	% barren	9.5	**	93.4	4.6	*	93.2	6.2	*	64.3	7.2	**	98.0
		biopolymeric C	0.4	ns	6.6	0.2	ns	6.8	2.7	ns	35.7	0.1	ns	2.0
	Tuscany	% barren	23.1	***	82.5	22.2	***	83.2	10.0	*	96.4	6.5	**	90.4
		biopolymeric C	1.7	ns	17.5	1.6	ns	16.8	0.2	ns	3.6	0.4	ns	9.6
	Sicily	% barren	18.0	**	62.7	13.0	**	63.9	13.5	**	66.1	16.3	**	58.6
		biopolymeric C	6.2	*	37.3	4.7	*	36.1	4.2	*	33.9	7.8	*	41.4
	Croatia	% barren	47.5	**	72.0	64.8	**	75.3	20.4	**	76.0	51.7	***	61.0
		biopolymeric C	4.7	*	28.0	4.0	ns	24.7	2.7	ns	24.0	11.5	**	39.0
	Montenegro	% barren	29.4	**	61.8	25.5	**	61.8	5.2	*	67.6	27.0	**	50.4
		biopolymeric C	8.6	**	38.2	8.0	*	38.2	2.0	ns	32.4	25.5	**	49.6
B)	Minorca	% barren	4.4	*	22.8	3.0	ns	22.2	6.8	*	27.7	9.5	**	18.2
		Chlorophyll-a	2.6	ns	15.6	2.0	ns	16.0	4.4	ns	20.8	7.9	*	16.4
		Phaeopigment	2.3	ns	14.2	1.8	ns	14.8	3.8	ns	18.9	7.1	*	15.5
		Protein	1.3	ns	8.5	1.4	ns	12.2	0.1	ns	0.9	0.3	ns	1.1
		Carbohydrate	0.3	ns	2.4	0.3	ns	2.9	0.1	ns	0.8	2.6	ns	7.8
		Lipid	0.1	ns	0.5	0.1	ns	0.5	0.3	ns	2.2	2.5	ns	7.6
		Chla to BPC %	7.8	**	32.7	3.9	*	27.4	5.6	*	24.5	3.5	ns	9.8
		Protein to BPC %	0.1	ns	1.1	0.2	ns	1.5	0.3	ns	2.3	4.0	ns	10.7
		Protein to carbohydrate	0.3	ns	2.1	0.3	ns	2.4	0.3	ns	1.9	5.3	*	12.9
	Sardinia	% barren	9.5	**	11.2	4.6	*	10.2	6.2	*	11.2	7.2	*	11.0
		Chlorophyll-a	13.0	***	12.9	10.4	***	16.4	13.9	**	17.1	7.7	**	11.4
		Phaeopigment	5.7	*	8.3	5.2	*	11.0	13.4	***	16.8	2.5	ns	5.3
		Protein	34.0	***	17.7	12.2	***	17.7	15.3	**	17.8	21.4	**	17.9
		Carbohydrate	1.8	ns	3.4	0.6	ns	1.8	0.2	ns	0.6	2.1	ns	4.5
		Lipid	4.7	*	7.3	2.8	ns	7.0	8.1	**	13.1	3.1	ns	6.3
		Chla to BPC %	14.2	**	13.4	7.6	**	13.9	4.4	ns	9.0	11.3	***	13.9
		Protein to BPC %	21.5	**	15.6	7.4	**	13.7	5.5	*	10.5	19.7	***	17.4
		Protein to carbohydrate	7.9	**	10.1	3.4	*	8.1	1.5	ns	3.8	8.7	**	12.2
	Tuscany	% barren	23.1	***	21.5	22.2	***	20.5	10.0	*	44.0	6.5	**	30.5
		Chlorophyll-a	2.3	ns	5.8	3.1	ns	7.1	0.1	ns	1.1	0.7	ns	5.3
		Phaeopigment	4.7	*	9.9	5.7	*	10.8	0.7	ns	6.1	1.6	ns	10.6
		Protein	3.0	ns	7.1	3.3	ns	7.3	0.4	ns	3.2	1.1	ns	7.4
		Carbohydrate	5.9	*	11.4	5.4	*	10.5	1.0	ns	8.2	0.9	ns	6.6
		Lipid	3.6	ns	8.2	2.9	ns	6.7	1.0	ns	8.1	0.7	ns	5.2
		Chla to BPC %	5.0	*	10.3	6.3	*	11.5	0.8	ns	6.2	1.2	ns	8.4
		Protein to BPC %	6.3	*	11.9	6.6	**	11.9	1.2	ns	9.4	1.7	ns	11.1
		Protein to carbohydrate	8.2	**	13.9	8.7	**	13.8	1.8	ns	13.6	2.4	ns	15.0
	Sicily	% barren	18.0	**	23.0	13.0	***	20.7	13.5	**	23.8	16.3	***	21.7
		Chlorophyll-a	12.5	**	19.9	10.1	**	18.4	8.3	*	18.7	11.5	***	18.8
		Phaeopigment	8.2	**	16.2	6.0	**	13.7	5.4	*	14.6	9.9	***	17.5
		Protein	11.7	**	19.3	8.1	**	16.4	9.1	*	19.8	12.0	**	19.1
		Carbohydrate	4.3	ns	10.8	3.6	*	9.8	2.5	ns	8.3	5.8	*	12.8
		Lipid	0.5	ns	1.8	0.7	ns	2.5	0.2	ns	0.7	1.1	ns	3.5
		Chla to BPC %	0.1	ns	0.2	0.2	ns	0.6	0.0	ns	0.1	0.2	ns	0.6
		Protein to BPC %	2.3	ns	6.7	4.2	*	10.9	4.2	*	12.3	0.9	ns	2.9
		Protein to carbohydrate	0.6	ns	2.0	2.4	ns	7.0	0.4	ns	1.7	0.9	ns	2.9
	Croatia	% barren	47.5	**	17.9	64.8	**	18.2	20.4	**	18.8	51.7	***	16.3
		Chlorophyll-a	14.5	**	12.8	13.3	**	12.0	6.3	*	10.8	39.5	***	15.5
		Phaeopigment	0.1	ns	0.3	0.1	ns	0.1	0.1	na	0.1	0.9	ns	1.6
		Protein	8.0	*	9.6	9.7	**	10.3	5.2	*	9.6	5.8	*	7.1
		Carbohydrate	8.9	**	10.1	7.8	**	9.2	5.1	*	9.4	20.8	***	13.1
		Lipid	0.3	ns	0.7	0.6	ns	1.2	1.3	ns	3.3	0.0	ns	0.1
		Chla to BPC %	21.6	**	14.8	27.0	***	15.3	9.4	**	13.6	26.3	***	14.1
		Protein to BPC %	32.8	**	16.6	36.4	***	16.4	13.7	**	16.2	48.9	***	16.1
		Protein to carbohydrate	40.3	***	17.3	48.8	***	17.4	18.0	**	18.0	48.4	**	16.1
	Montenegro	% barren	29.4	**	21.4	25.5	**	20.5	5.2	ns	12.6	27.0	***	21.6
		Chlorophyll-a	0.8	ns	2.2	1.0	ns	2.6	0.5	ns	1.6	2.5	ns	5.9
		Phaeopigment	11.9	***	15.6	11.0	**	15.0	2.7	ns	7.8	7.8	*	13.0
		Protein	3.6	*	7.7	4.0	*	8.1	9.5	**	17.8	1.1	ns	2.9
		Carbohydrate	14.7	**	17.1	13.8	**	16.6	4.2	ns	10.9	34.1	***	22.9
		Lipid	0.9	ns	2.3	1.0	ns	2.5	0.5	ns	1.8	3.6	ns	7.8
		Chla to BPC %	1.4	ns	3.6	1.7	ns	4.2	2.9	ns	8.2	0.4	ns	1.2
		Protein to BPC %	13.4	***	16.4	13.8	***	16.6	13.3	**	21.0	7.4	*	12.6
		Protein to carbohydrate	9.0	**	13.6	9.8	**	14.1	9.9	**	18.3	6.9	*	12.1

Figure S1. Average (±SE) multivariate dispersion (PERMDISP) based on dissimilarity matrices of sedimentary organic matter biochemical compounds and composition at the scales of area and state.

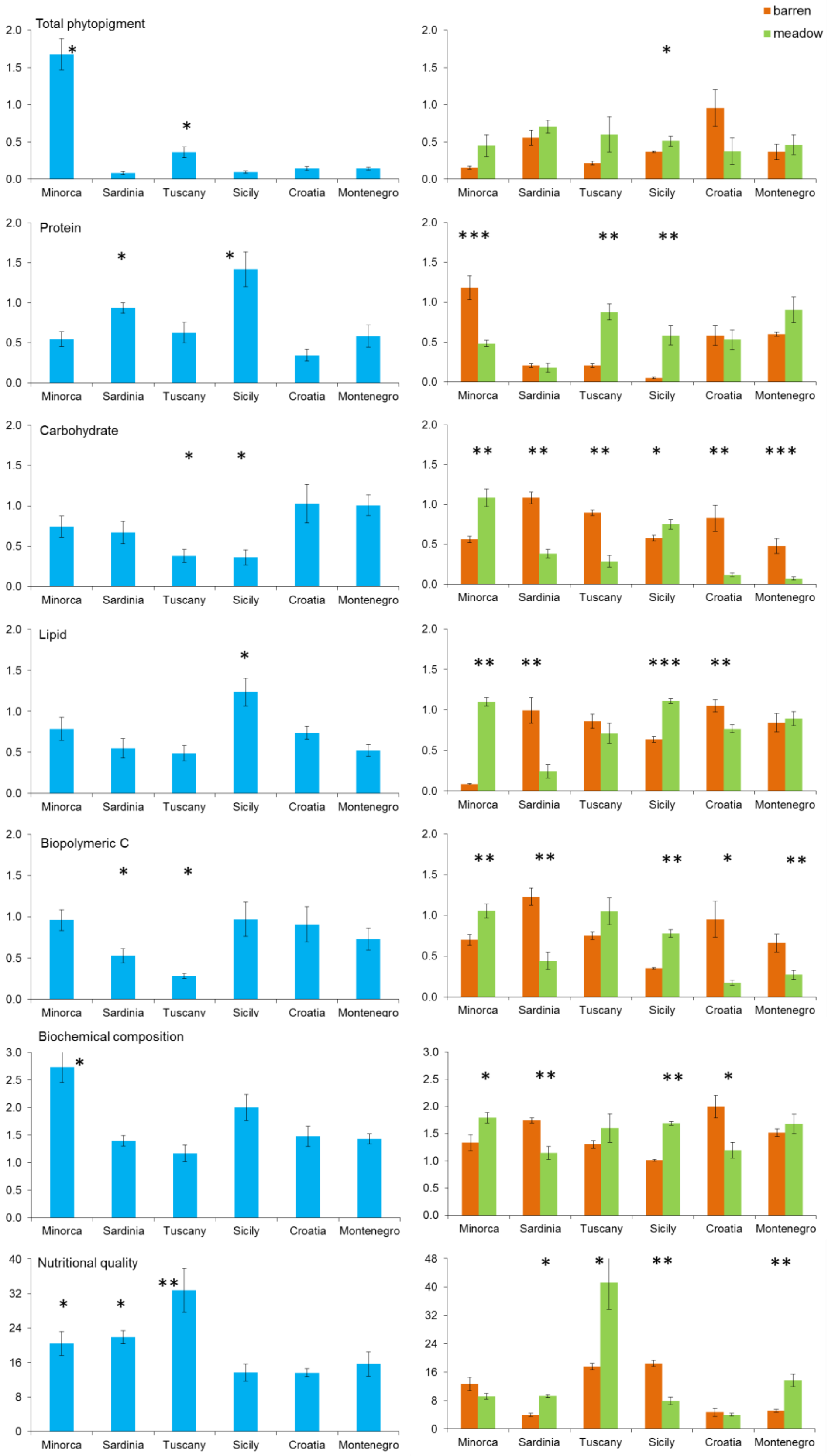

Figure S2. Average (±SE) multivariate dispersion (PERDISP) based on dissimilarity matrices of meiofaunal abundance, biomass, taxonomic composition and nematode species composition at the scales of area and state.

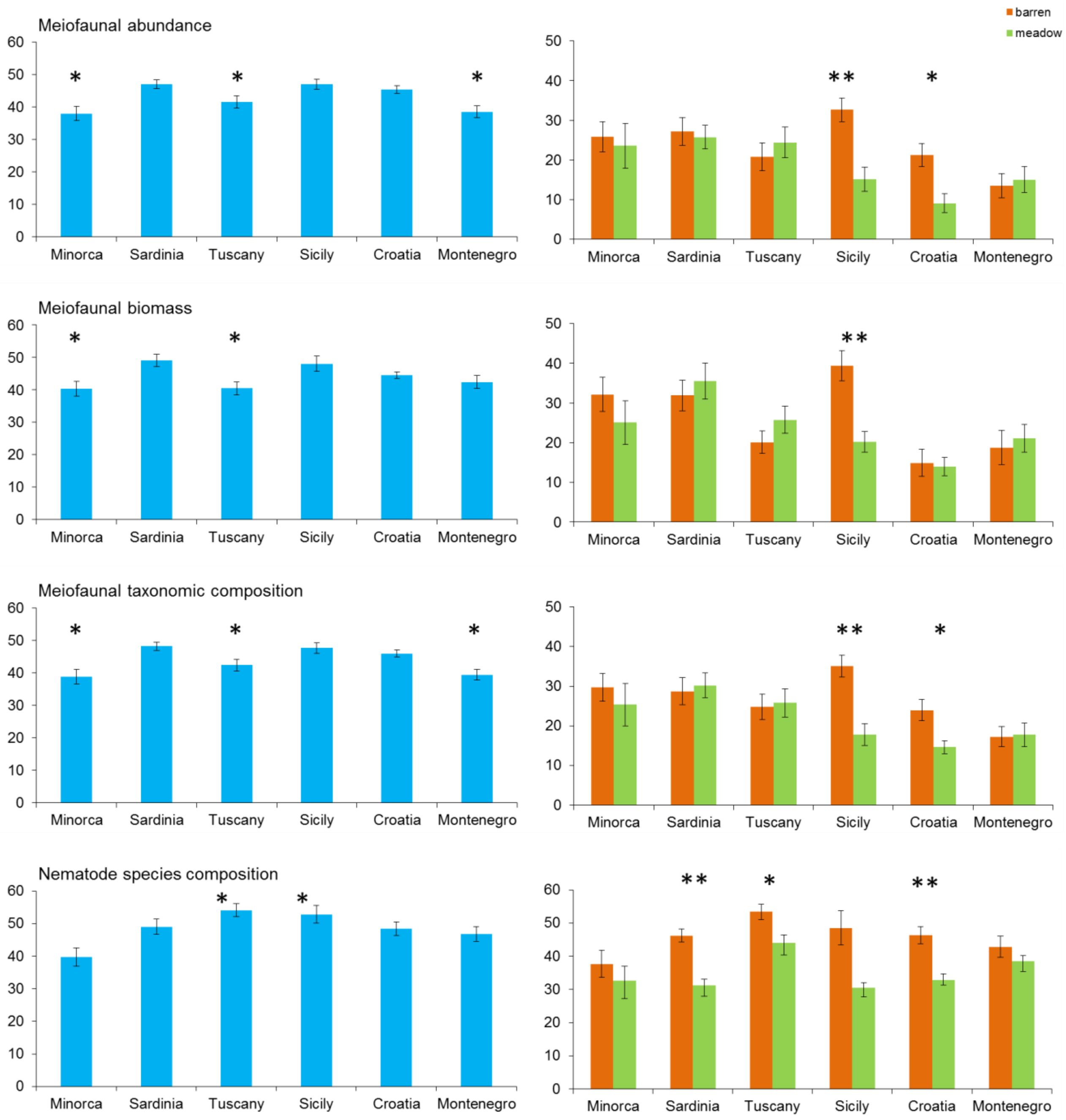

Figure S3. Effect of the presence of barrens on the concentration of sedimentary organic matter biochemical compounds.

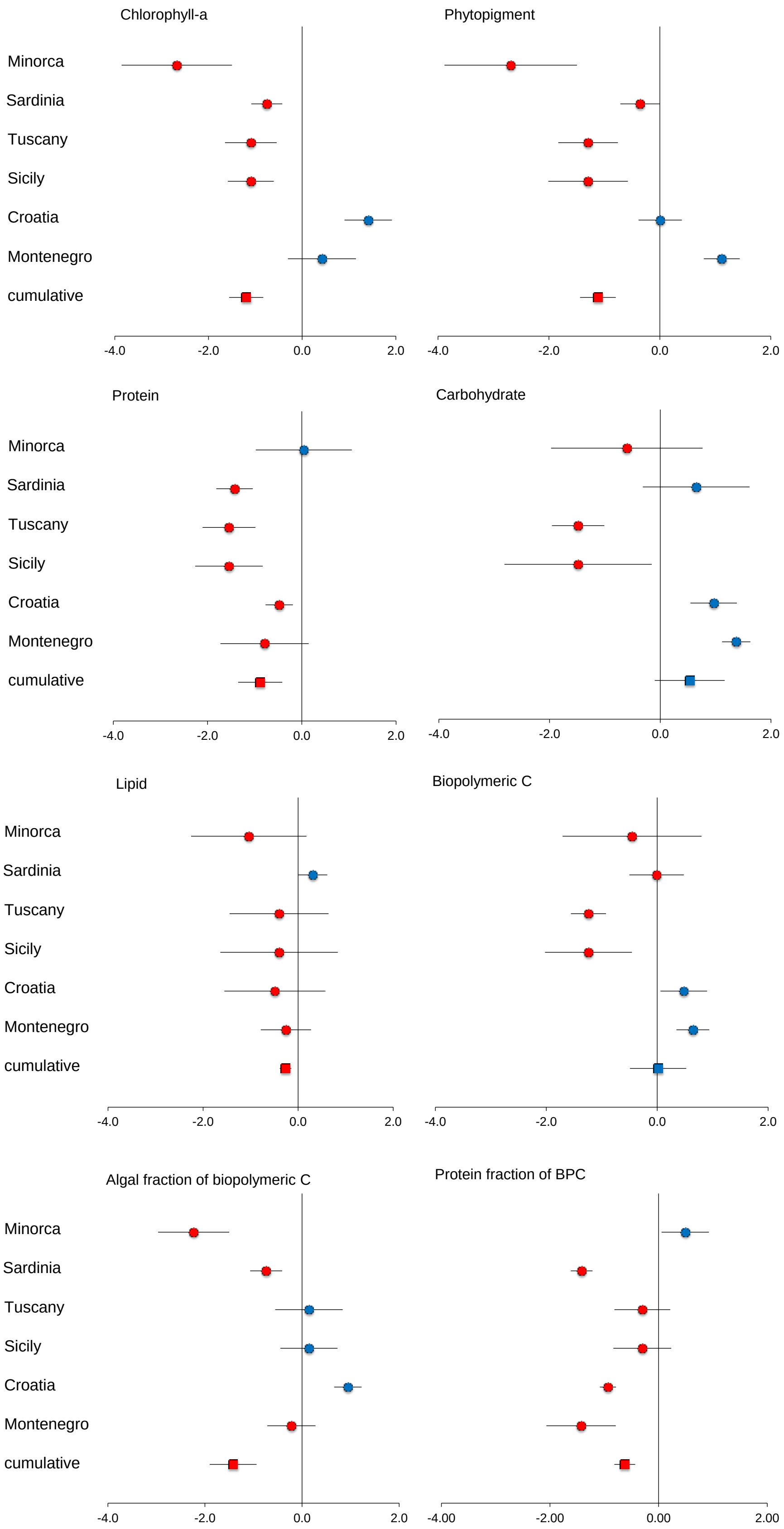

Supplement: Supplementary Information [file srep34544-s1.pdf]
